# Dog leucocyte antigen (DLA) class II haplotypes and risk of canine diabetes mellitus in specific dog breeds

**DOI:** 10.1186/s40575-020-00093-9

**Published:** 2020-10-31

**Authors:** A. L. Denyer, J. P. Massey, L. J. Davison, W. E. R. Ollier, B. Catchpole, L. J. Kennedy

**Affiliations:** 1 Department of Pathology and Pathogen Biology, Royal Veterinary College, Hatfield, UK; 2 Centre for Integrated Genomic Medical Research, University of Manchester, Stopford Building, Oxford Road, Manchester, M13 9PT UK; 3 Wellcome Centre for Human Genetics, University of Oxford, Oxford, UK; 4 Department of Clinical Sciences and Services, Royal Veterinary College, Hatfield, UK

**Keywords:** Diabetes mellitus, Canine, Genetics, Dog leucocyte antigen

## Abstract

**Background:**

Canine diabetes mellitus (DM) is a common endocrine disease in domestic dogs. A number of pathological mechanisms are thought to contribute to the aetiopathogenesis of relative or absolute insulin deficiency, including immune-mediated destruction of pancreatic beta cells. DM risk varies considerably between different dog breeds, suggesting that genetic factors are involved and contribute susceptibility or protection. Associations of particular dog leucocyte antigen (DLA) class II haplotypes with DM have been identified, but investigations to date have only considered all breeds pooled together. The aim of this study was to analyse an expanded data set so as to identify breed-specific diabetes-associated DLA haplotypes.

**Methods:**

The 12 most highly represented breeds in the UK Canine Diabetes Register were selected for study. DLA-typing data from 646 diabetic dogs and 912 breed-matched non-diabetic controls were analysed to enable breed-specific analysis of the DLA. Dogs were genotyped for allelic variation at DLA-DRB1, -DQA1, -DQB1 loci using DNA sequence-based typing. Genotypes from all three loci were combined to reveal three-locus DLA class II haplotypes, which were evaluated for statistical associations with DM. This was performed for each breed individually and for all breeds pooled together.

**Results:**

Five dog breeds were identified as having one or more DLA haplotype associated with DM susceptibility or protection. Four DM-associated haplotypes were identified in the Cocker Spaniel breed, of which one haplotype was shared with Border Terriers. In the three breeds known to be at highest risk of DM included in the study (Samoyed, Tibetan Terrier and Cairn Terrier), no DLA haplotypes were found to be associated with DM.

**Conclusions:**

Novel DLA associations with DM in specific dog breeds provide further evidence that immune response genes contribute susceptibility to this disease in some cases. It is also apparent that DLA may not be contributing obvious or strong risk for DM in some breeds, including the seven breeds analysed for which no associations were identified.

## Plain English summary

Diabetes mellitus (DM) affects approximately 1 in 300 dogs in the UK and is much more common in some breeds than others. Multiple factors and biological processes are involved in canine DM. There is preliminary evidence that genetic factors may explain why some breeds are predisposed to, or protected from, developing this disease. Previous evidence has indicated that the immune system may contribute to destruction of the pancreas in diabetic dogs, similar to that seen in human type 1 DM. A collection of genes encoding the dog leucocyte antigens (DLA) play a critical role in regulating immune responses and previous studies have implicated a role for these genes in canine DM. However, no investigations have yet been conducted in individual pedigree dog breeds. This study compared variation within the DLA genes of diabetic and non-diabetic dogs for 12 different breeds. In 5 breeds, increased risk for or protection from DM was associated with particular DLA gene variations. However, no associations were identified in the three breeds at highest risk of DM included in the study (Samoyed, Tibetan Terrier and Cairn Terrier). This study supports a role for DLA-encoded immune regulation in the development of DM in certain dog breeds. Future studies of gene function in single breeds will help identify causal variants and mechanisms, which may lead to improved diagnosis and management of affected dogs.

## Background

Canine DM is a relatively common endocrinopathy encountered in routine veterinary clinical practice, with a UK prevalence of 0.32–0.34% [1, 2]. Cases are diagnosed on the basis of persistent fasting hyperglycaemia, persistent glycosuria and compatible clinical signs [3]. Virtually all diabetic dogs require daily insulin injections [3], although the precise cause of the pancreatic beta cell dysfunction/destruction remains elusive.

Most cases of canine DM are diagnosed in dogs aged between 5 and 12 years of age [1]. A classification system has been proposed, based on the underlying pathogenesis of the hyperglycaemia, namely either insulin deficiency diabetes (IDD) or insulin resistance diabetes (IRD) [4]. Further classification nomenclature has recently been proposed by the European Society for Veterinary Endocrinology (ESVE) project ALIVE (Agreeing Language in Veterinary Endocrinology) [5]. Apart from female entire dogs, which can develop a form of IRD during dioestrus or pregnancy, most cases of canine DM can be classified as IDD [4]. However, the underlying pathogenesis of insulin deficiency is likely to be complex and heterogeneous. DM is likely to be the consequence of a decline in pancreatic beta cell mass, resulting from increased beta cell destruction and/or impaired beta cell regeneration. Histopathological studies have reported reduced populations of beta cells in the pancreatic tissue of diabetic dogs [6, 7]. This has prompted research into whether beta cell loss occurs due to immune-mediated destruction, as is the case in human Type 1 DM (T1D) [8]. The most common autoantibodies identified in human T1D (against insulin, GAD-65 and IA-2) have been detected in a small proportion of diabetic dogs [9–12]; other studies have failed to detect autoantibodies [13]. Lymphocytic infiltration of pancreatic islets, as seen at the onset of human T1D [14, 15], has rarely been reported in dogs [7, 13]. Therefore, the role of the adaptive immune system in canine DM remains uncertain. It has also been proposed that canine DM might be more similar to human latent autoimmune diabetes of adults (LADA) rather than T1D [16]. This is a more slowly progressive immune-mediated destruction of pancreatic beta cells occurring in combination with other non-immunological risk factors, leading to reduced beta cell mass and insulin deficiency later in life.

The striking pattern of breed predispositions for canine DM strongly suggests that genetic risk factors contribute to disease susceptibility. Breeds such as the Samoyed, Yorkshire Terrier, Australian Terrier and Miniature Schnauzer have all been reported to be at high risk in independent studies, whereas the Boxer, Golden Retriever and German Shepherd Dog are considered to be at low risk of developing DM [1, 2, 17, 18]. A large number of diabetes susceptibility genes, each contributing relatively small effects, have been identified in human T1D [19], Type 2 DM (T2D) [20] and monogenic DM [21]. This has prompted candidate gene studies in canine DM, which have largely focussed on T1D genes involved with immune function [22–25] and human monogenic diabetes genes [26].

Genes encoded within the major histocompatibility complex (MHC) play critical roles in the regulation of the adaptive immune response in higher species. These roles include their involvement in the setting of baseline immune tolerance, allowing distinction of ‘self’ antigens from ‘non-self’ antigens, and also influencing the degree of immune response that develops to particular antigens. A striking feature of many of MHC loci is their extreme levels of gene polymorphism. Certain MHC class II gene polymorphisms are highly associated with the development of autoimmune conditions and other immune-mediated diseases in humans [27, 28] and other mammals including dogs [29, 30].

Canine MHC genes, known as the dog leucocyte antigen (DLA) genes are found on chromosome 12. There are four functional class II MHC genes in the dog: DLA-DRA1, -DRB1, -DQA1 and -DQB1 [31]. These encode cell-surface heterodimers which bind antigenic peptides in a peptide binding groove and present these to T cell receptors for regulation of the adaptive immune response. DLA-DRA1 appears to be monomorphic, while the other three functional class II genes are highly polymorphic, resulting in diversity in the antigen binding capabilities of the class II molecules. The extensive linkage disequilibrium in this region results in particular combinations of alleles being found together as preferential haplotype combinations. Therefore, three-locus DLA class II haplotypes of DLA-DRB1, -DQA1 and -DQB1 are often used to study the relationship of the DLA to disease.

Just as some human HLA gene polymorphisms and haplotype combinations are present or absent in some ethnic groups, or are seen at different gene frequencies in different populations, the same is observed across different pedigree dog breeds, but to even more extreme levels [32, 33]. Furthermore, specific HLA-types influencing disease risk vary between different human ethnic populations [34, 35]. A previous candidate gene study, investigating the role of the MHC in canine DM, identified four DLA haplotypes that were associated with disease risk or protection [25]. That study compared DLA class II alleles and haplotypes in pooled populations of dogs with canine DM to unaffected dogs, but did not have sufficient sample sizes to enable analysis by individual breed. However, pooling of data between breeds can obscure differences in DM pathogenesis that may exist among breeds. Therefore, studying the role of DLA haplotypes within individual breeds can improve understanding of the role of DLA genes in particular breeds and help to identify those cases where underlying pathology may be similar.

The aim of the current study was to determine whether specific DLA haplotypes are associated with risk or protection from DM in individual dog breeds and whether combined analysis of all 12 breeds would support the findings of the previous study. Identifying whether DLA genes influence the risk of canine DM will improve understanding of whether this endocrinopathy is likely to have an immune-mediated pathogenesis in certain breeds.

## Methods

### Study population

EDTA blood samples from UK diabetic dogs, submitted by veterinary surgeons to the Royal Veterinary College (RVC) Canine Diabetes Register with informed consent (Ethics Review Board number: URN 2017 1685–3), were used for this study. A diagnosis of DM was made on the basis of persistent hyperglycaemia, glucosuria and consistent clinical signs. Dogs were either newly diagnosed or had been receiving insulin treatment for a variable period of time. Following diagnostic testing, residual blood samples were made available for research. Samples from female entire diabetic dogs were excluded, as these were potentially affected with dioestrus diabetes. Where age data were available, diabetic dogs < 6 months old at diagnosis were also excluded as these were potentially affected with a form of juvenile DM. Blood samples from non-diabetic dogs were obtained from the RVC DNA Archive and the Companion Animal DNA Archive (University of Manchester). Control samples with a recorded clinical history of autoimmune disease were also excluded.

To facilitate breed-specific analysis, 12 different dog breeds were selected in which there were samples from at least 20 cases, and where the number of controls was equal to or greater than the number of cases. The sample size for each breed included in the study, and the published odds ratio for UK population-based breed-associated risks for DM [36], are summarised in Table 1.

**Table 1:** DM risk for the specific breeds analysed [36] and samples sizes

Breed	Case (*n*)	Control (*n*)	Published breed risk of DM (odds ratio)	95% Confidence interval
Samoyed	43	70	35.84	25.58–50.22
Tibetan Terrier	20	40	10.39	7.28–14.83
Cairn Terrier	30	30	9.76	7.27–13.09
Miniature Schnauzer	28	38	3.62	2.57–5.09
WHWT	133	150	3.04	2.55–3.63
Border Collie	57	87	2.02	1.64–2.5
Border Terrier	27	27	1.8	1.26–2.58
Labrador Retriever	111	240	1.67	1.43–1.94
Yorkshire Terrier	45	45	1.55	1.25–1.93
CKCS	48	48	1.41	1.08–1.85
Cocker Spaniel	55	80	1.25	0.97–1.6
JRT	49	57	0.61	0.48–0.77
Total (n)	646	912		

Breeds are sorted based on the reported risk of DM [36]. *n* = number of dogs, *CKCS* Cavalier King Charles Spaniel, *JRT* Jack Russell Terrier, *WHWT* West highland White Terrier

### DLA class II typing

Dogs were genotyped for three DLA class II loci: DLA-DRB1, DLA-DQA1 and DLA-DQB1. The DLA data used in a previous study [25] was also included in the present study. The DLA-typing methods and primers used for these samples are described in detail elsewhere [25]. The additional samples used in this study were similarly analysed by DNA sequence-based typing of exon 2 for each DLA locus, performed using a touchdown PCR protocol. Primers are listed in Supplementary Table 1 [37–39]. Purification of PCR products was performed (ExoSAP-IT PCR Clean-up Reagent, Applied Biosystems or GenElute PCR Clean-Up Kit, Sigma-Aldrich) prior to DNA sequencing using a standard Sanger sequencing protocol. Sequencing data were analysed by either SBTengine (GenDx, Genome Diagnostics B.V.) or MatchTools/MatchTools Navigator (Applied Biosystems). DNA sequences were aligned to a consensus sequence and each polymorphic site analysed electronically or manually. Sequences were electronically aligned against a reference sequence library to assign the correct allele for each locus. DLA haplotypes were subsequently assigned, based on established DLA-types for each breed [40].

### Statistical analysis

Haplotype counts were performed in Microsoft Excel for cases and controls of each breed. Statistical analysis was carried out using R software (R Foundation for Statistical Computing). Contingency Tables were used to calculate odds ratios; confidence intervals were determined using Fisher’s Exact Test. Odds ratios were considered to be significant when the upper and lower confidence intervals did not cross 1.

## Results

DLA haplotype frequencies observed in each breed are summarised in Table 2 (higher risk breeds) and Table 3 (lower risk breeds). For ease of reference, haplotype IDs were arbitrarily assigned to the 30 most common haplotypes observed in the study (Supplementary Table 2). The two most common DLA haplotypes observed in the study population were haplotype 1 (DRB1*001:01--DQA1*001:01--DQB1*002:01; 13.6% cases vs. 12.1% controls) and haplotype 6 (006:01--005:01:1--007:01; 14.1% cases vs. 10.9% controls). These were also two of the three most commonly observed haplotypes in the previous study [25].

**Table 2:** Haplotype frequencies (%) observed in the six highest risk breeds included in the study

Haplotype ID	Haplotype			Samoyed		Tibetan Terrier		Cairn Terrier		Miniature Schnauzer		WHWT		Border Collie	
	DRB1	DQA1	DQB1	Cases ***n*** = 43	Cntr ***n*** = 70	Cases ***n*** = 20	Cntr ***n*** = 40	Cases ***n*** = 30	Cntr ***n*** = 30	Cases ***n*** = 28	Cntr ***n*** = 38	Cases ***n*** = 133	Cntr ***n*** = 150	Cases ***n*** = 57	Cntr ***n*** = 87
1	001:01	001:01	002:01			2.5	5.0	5.0	10.0		3.9	39.5	39.7	6.1	3.4
2	001:01	001:01	036:01			17.5	11.3			21.4	14.5	**0**	**2.0**	2.6	2.9
3	001:01	009:01	001:01									21.8	25.0		
4	002:01	009:01	001:01						3.3					16.7	4.9
9	009:01	001:01	008:01:1							58.9	64.5			4.4	
10	009:01	001:01	008:02	23.5	17.1									3.5	4.0
15	013:01	001:01	002:01							8.9	6.6			12.3	21.8
16	015:01	006:01	003:01		3.6				8.3						
17	015:01	006:01	011:01					3.3				7.1	7.7		
18	015:01	006:01	019:01–054:02	63.2	65.0										
19	015:01	006:01	020:02						3.3			17.7	14.3		
21	015:01	006:01	023:01			15.0	11.3	43.3	35.0			6.8	4.3	24.6	17.8
22	015:01	006:01	026:01			7.5	18.8								
23	015:01	009:01	001:01									4.1	3.7		
24	015:02	006:01	003:01								2.6				
25	015:02	006:01	023:01			17.5	15.0	35.0	21.7	3.6	6.6			3.5	4.6
26	018:01	001:01	002:01											7.9	9.6
28	020:01	004:01	013:03	7.4	10.0										
29	023:01	003:01	005:01							3.6				7.9	6.9
30	079:01	001:01	002:01			30.0	26.3								
	001:01	002:01	036:01			2.5									
	003:01	001:01	008:01:1			2.5	5.0	10.0	13.3						
	015:02	006:01	054:01			2.5									
	040:01	010:01	019:01	4.4	3.6										
	112:01	001:01	008:01:2			2.5	3.8								

Only haplotypes with a frequency > 2% are shown. Haplotypes which were significantly different between cases and controls in an individual breed are **shown in bold**. Haplotypes are sorted by haplotype ID, where these were assigned (Supplementary Table 2; assigned to the 30 most commonly observed haplotypes for ease of reference throughout the study). Breeds are listed in order of breed risk for DM, as indicated in Table 1. *n* = number of dogs, *Cntr* Controls, *WHWT* West highland White Terrier

**Table 3:** Haplotype frequencies (%) observed for the six lowest risk breeds included in the study

Haplotype ID	Haplotype			Border Terrier		Labrador Retriever		Yorkshire Terrier		CKCS		Cocker Spaniel		JRT	
	DRB1	DQA1	DQB1	Cases ***n*** = 27	Cntr ***n*** = 27	Cases ***n*** = 111	Cntr ***n*** = 240	Cases ***n*** = 45	Cntr ***n*** = 45	Cases ***n*** = 48	Cntr ***n*** = 48	Cases ***n*** = 55	Cntr ***n*** = 80	Cases ***n*** = 49	Cntr ***n*** = 57
1	001:01	001:01	002:01			**20.3**	**13.8**							13.3	14.0
2	001:01	001:01	036:01											8.2	7.9
4	002:01	009:01	001:01	57.4	50.0			7.8	12.2					8.2	7.9
5	005:01	003:01	005:01	11.1	7.4				2.2					3.1	2.6
6	006:01	005:01:1	007:01			14.4	12.1	45.6	37.8	3.1	4.2	**89.1**	**56.3**	6.7	7.0
7	006:01	005:01:1	020:01			3.2	3.5			5.2		**8.2**	**23.1**		
8	008:02	003:01	004:01			3.2	3.5							2.0	
9	009:01	001:01	008:01:1							16.7	20.8			9.2	6.1
11	011:01	002:01	013:02	13.0										2.0	2.6
12	011:01	002:01	013:03							37.5	51.0		2.5		
14	012:01	004:01	017:01–013:03			26.6	32.7					**0**	**5.0**		
15	013:01	001:01	002:01					5.6						9.2	8.8
16	015:01	006:01	003:01											2.0	2.6
19	015:01	006:01	020:02	**7.4**	**27.8**			3.3	3.3			**0**	**6.9**	2.0	
20	015:01	006:01	022:01					3.3	7.8					7.1	4.4
21	015:01	006:01	023:01			7.7	9.0		3.3					5.1	7.0
25	015:02	006:01	023:01			13.5	14.4	15.6	16.7		3.1				3.5
26	018:01	001:01	002:01											2.0	
27	018:01	001:01	008:02	3.7										6.1	7.0
28	020:01	004:01	013:03			2.3	2.5			**35.4**	**16.7**				
29	023:01	003:01	005:01					4.4	4.4						
	001:01	002:01	036:01											2.0	
	001:02	001:01	002:01											2.0	
	002:02	009:01	001:01					4.4						2.0	
	005:01	009:01	023:01		3.7										
	006:01	005:01:1	028:01					6.7							
	008:03	003:01	004:01	3.7											
	012:01	004:01	013:03			2.7									
	017:01	002:01	013:03						2.2						
	020:01	004:01	013:01								2.1				
	075:01	004:01	013:03						2.2						

Only haplotypes with a frequency > 2% are shown. Haplotypes which were significantly different between cases and controls in an individual breed are **shown in bold**. Haplotypes are sorted by haplotype ID, where these were assigned (Supplementary Table 2; assigned to the 30 most commonly observed haplotypes for ease of reference throughout the study). Breeds are listed in order of breed risk for DM, as indicated in Table 1. *n* = number of dogs, *Cntr* controls, *CKCS* Cavalier King Charles Spaniel, *JRT* Jack Russell Terrier

The frequency of DLA haplotypes in cases and controls was analysed separately for individual breeds. Eight DLA haplotypes were significantly different comparing cases and controls, across five breeds (Table 4). Four haplotypes (IDs: 6, 7, 14 & 19) in the Cocker Spaniel breed were associated with DM susceptibility or protection. Haplotype 6 (DLA 006:01--005:01:1--007:01) was associated with DM risk (OR = 6.3, 95% CI: 3.1–13.7). In contrast, haplotype 7 (006:01--005:01:01--020:01), which only differs from risk haplotype 6 at the DQB1 locus, was associated with protection against DM (OR = 0.30, 95% CI = 0.1–0.7). Similarly, haplotype 1 (001:01--001:01--002:01), which was associated with increased DM risk in Labrador Retrievers, only differs at the DQB1 locus from haplotype 2 (001:01--001:01--036:01), which was associated with lower DM risk in West Highland White Terriers (WHWT).

**Table 4:** Breed-specific analysis (only significant haplotypes and breeds shown)

Breed (*n* cases/*n* controls)	Haplotype ID	Haplotype			Cases %	Controls %	Odds ratio	95% Confidence interval	Protective/Risk	Number of other breeds carrying haplotype
		DRB1	DQA1	DQB1						
Border Terrier (27/27)	19	015:01	006:01	020:02	7.4	27.8	0.211	0.05–0.73	Protective	6
CKCS (48/48)	28	020:01	004:01	013:03	35.4	16.7	2.73	1.33–5.81	Risk	5
Cocker Spaniel (55/80)	6	006:01	005:01:1	007:01	89.1	56.3	6.31	3.14–13.65	Risk	6
	7	006:01	005:01:1	020:01	8.2	23.1	0.297	0.12–0.66	Protective	3
	14	012:01	004:01	017:01–013:03	0.0	5.0	N/A	0–0.83	Protective	4
	19	015:01	006:01	020:02	0.0	6.9	N/A	0–0.56	Protective	6
Labrador Retriever (111/240)	1	001:01	001:01	002:01	20.3	13.8	1.59	1.02–2.47	Risk	7
WHWT (133/150)	2	001:01	001:01	036:01	0.0	2.0	N/A	0–0.95	Protective	7

DLA class II haplotypes significantly associated (upper and lower 95% confidence intervals did not cross 1) with diabetes mellitus in individual breeds, calculated using Fisher’s exact test. *n* = number of dogs, *CKCS* Cavalier King Charles Spaniel, *WHWT* West highland White Terrier, *N/A* Not applicable

Haplotype 19 (015:01--006:01--020:02) was found to be a DM protective haplotype in both the Cocker Spaniel and Border Terrier breeds. No other DLA haplotypes were associated with risk or protection in more than one breed. Furthermore, no risk or protective haplotypes were identified for the three breeds at greatest risk of DM included in the study: the Samoyed, Tibetan Terrier and Cairn Terrier.

All of the haplotypes identified as having some association with DM in an individual breed were also carried by multiple other breeds included in this study (Table 4). The most common haplotype in the Cocker Spaniel breed (haplotype 6; 006:01--005:01:1--007:01) was also found in all other breeds, apart from the four breeds with the highest risk of developing DM (Samoyed, Cairn Terrier, Tibetan Terrier and Miniature Schnauzer) and the Border Terrier. This haplotype is common among the general dog population, being found in 70% of dog breeds, albeit at a wide range of frequencies (L.J. Kennedy; personal communication).

A combined analysis of all 12 breeds was performed, in order to compare findings more directly with the observations made in Kennedy et al. (2006). This analysis revealed four haplotypes associated with DM risk or protection (Table 5). Three of these haplotypes had been associated with DM risk/protection in the individual analysis of the Cocker Spaniel breed (IDs 6, 7 and 14; Table 4). The remaining haplotype from the combined analysis was not associated with DM risk in any individual breeds. None of the haplotypes from the combined analysis had been significantly associated with risk of DM in the previous study [25], in which a smaller number of dogs, representing a wider range of breeds, had been examined together.

**Table 5:** Combined analysis of all 12 breeds (only significant haplotypes shown)

Haplotype ID	Haplotype			Cases %	Controls %	Odds ratio	95% Confidence interval	Protective/Risk
	DRB1	DQA1	DQB1					
6	006:01	005:01:1	007:01	14.1	10.9	1.34	1.07–1.67	Risk
7	006:01	005:01:1	020:01	1.7	3.0	0.557	0.32–0.93	Protective
14	012:01	004:01	017:01–013:03	4.5	9.3	0.469	0.34–0.64	Protective
16	015:01	006:01	003:01	0.3	1.2	0.267	0.07–0.79	Protective

DLA class II haplotypes significantly associated (upper and lower 95% confidence intervals did not cross 1) with diabetes mellitus in combined analysis with all breeds pooled together, calculated using Fisher’s exact test

### Analysis of high-risk breeds

Given that no haplotypes were identified as significantly different between diabetic cases and non-diabetic controls for the breeds at highest risk of canine DM (Samoyed, Tibetan Terrier and Cairn Terrier), the DLA profiles for these high-risk breeds were examined in order to identify any breed-associated patterns that may relate to their predisposition to DM. The DLA haplotypes observed in each of these three breeds are shown in Table 6.

**Table 6:** DLA haplotypes identified in cases and controls for the three highest-risk breeds included in the study

Haplotype ID	Haplotype			Samoyed		Tibetan Terrier		Cairn Terrier		Comment
	DRB1	DQA1	DQB1	Cases % *2n* = 68	Controls % *2n* = 140	Cases % *2n* = 40	Controls % *2n* = 80	Cases % *2n* = 60	Controls % *2n* = 60	
1	001:01	001:01	002:01			2.5	5.0	5	10	Risk haplotype in Labrador Retriever
2	001:01	001:01	036:01			17.5	11.3			
4	002:01	009:01	001:01					0	3	
9	009:01	001:01	008:01:1			2.5	5.0	10	13	
10	009:01	001:01	008:02	23.5	17.1					
12	011:01	002:01	013:03					0	2	
14	012:01	004:01	017:01–013:03					0	2	Protective haplotype in combined analysis and in Cocker Spaniel
16	015:01	006:01	003:01	0	3.6			0	8	Protective haplotype in combined analysis
17	015:01	006:01	011:01	1.5	0			3	0	
18	015:01	006:01	019:01–054:02	63.2	65					Unique to Samoyed
19	015:01	006:01	020:02					0	3	
21	015:01	006:01	023:01			15.0	11.3	43	35	
22	015:01	006:01	026:01			7.5	18.8			Unique to Tibetan Terrier
24	015:02	006:01	003:01			0.0	1.3			
25	015:02	006:01	023:01	0	0.7	17.5	15.0	35	22	
26	018:01	001:01	002:01					2	0	
27	018:01	001:01	008:02					0	2	
28	020:01	004:01	013:03	7.4	10					Risk haplotype in CKCS
30	079:01	001:01	002:01			30.0	26.3			Unique to Tibetan Terrier
	001:01	002:01	036:01			2.5	0			
	015:02	006:01	054:01			2.5	0			Unique to Tibetan Terrier cases
	015:03	006:01	013:05			0	1.3			Unique to Tibetan Terrier controls
	015:03	006:01	023:01			0	1.3			Unique to Tibetan Terrier controls
	017:01	002:01	013:03					2	0	
	040:01	010:01	019:01	4.4	3.6					Unique to Samoyed
	112:01	001:01	008:01:2			2.5	3.8			Unique to Tibetan Terrier

The Samoyed breed was found to have a relatively restricted DLA profile, with only seven different haplotypes. Haplotype 18 (015:01--006:01--019:01--054:02), which carries two alleles at the DQB1 locus, was only found in the Samoyed dogs in this study and accounted for more than half of the haplotypes identified in both cases (63.25%) and controls (65%) of this breed. A second haplotype, (040:01--010:01--019:01, no haplotype ID) was also found only in Samoyeds, although at a relatively low frequency (4.4% cases, 3.6% controls). The second most common haplotype in Samoyeds, haplotype 10 (009:01--001:01--008:02) differs by a single non-synonymous base-pair change from haplotype 9 (009:01--001:01--008:01:1), which was also observed in the two other highest risk breeds.

The Tibetan and Cairn Terriers were found to have a more diverse DLA profile than Samoyeds, with 13 different haplotypes being identified in each breed. Six haplotypes were identified exclusively within the Tibetan Terrier breed. All of these were found at low frequency except haplotype 22 (015:01-- 006:01--026:01), which represented 7.5% of diabetic and 18.8% of control haplotypes, and haplotype 30 (079:01--001:01--002:01) which represented 30% of diabetic and 26.3% of control haplotypes. Haplotype 1 (001:01--001:01--002:01), which was found to confer diabetes risk in Labrador Retrievers (Table 4), was also identified in Tibetan and Cairn Terriers. However, this was one of the most common haplotypes observed in the study. No haplotypes were identified exclusively within the Cairn Terrier breed.

## Discussion

The primary aim of this study was to identify whether DLA class II haplotypes are associated with risk or protection from DM in individual UK dog breeds. A secondary aim was to determine whether a pooled analysis of all 12 breeds included in the current study would support the findings of the previous study by Kennedy et al. (2006). Our study indicated that no DLA haplotypes were associated with risk of DM in the ‘highest DM risk’ breeds (Samoyed, Cairn Terrier, Tibetan Terrier and Miniature Schnauzer). Five ‘moderate DM risk’ breeds were found to possess DLA haplotypes that were associated with risk or protection from DM; all of these haplotypes were also present in other breeds, although only one DLA haplotype was associated with protection from DM in more than one breed (haplotype 19; DLA-DRB1*015:01--DQA1*006:01--DQB1*020:02, found to confer protection in Cocker Spaniel and Border Terrier breeds). These findings provide further evidence for the role of MHC class II and the adaptive immune system in the pathogenesis of canine DM in some breeds and suggest that the disease is likely to be heterogeneous among breeds.

As a result of its popularity, the Labrador Retriever was the most highly represented breed in this study (111 cases / 240 controls). It is a breed at a moderate [36] to neutral [2] risk of developing DM. In this breed, DLA haplotype 1 (001:01--001:01--002:01) was found to be associated with increased risk of DM (OR = 1.59, 95% CI = 1.02–2.47), which suggests that this particular DLA-type might play a role in the pathogenesis of beta cell loss/dysfunction in this breed. This DLA haplotype has previously been associated with increased risk of hypothyroidism in Gordon Setters [41] as well as primary hypoadrenocorticism (Addison’s disease; AD) in WHWT and Leonberger breeds [42]. Notably, both hypothyroidism associated with lymphocytic thyroiditis and primary hypoadrenocorticism are considered to have an autoimmune basis and demonstrate breed differences in susceptibility [43, 44]. Additionally, the DLA-DRB1*001:01 allele has been found to confer risk of anal furunculosis in GSDs [45, 46], suggesting that this particular DRB1 allele may be important in dysregulated inflammatory disease processes.

In the second most highly represented breed, the WHWT (133 cases/150 controls), which is also the breed at fifth highest risk of DM in this study, DLA haplotype 2 (001:01--001:01--036:01) was associated with protection from DM (95% CI = 0–0.95). This haplotype was identified in 6 controls (2% of controls) and not in any diabetic dogs, which supports a role for this haplotype in protecting against DM in this breed. However, because it was only identified in only 2% of controls and the 95% CI is very wide (0–0.95), this association should be interpreted with caution and further investigated in a larger cohort of WHWTs. A similar observation was made by a study of DLA class II haplotypes in AD [42]: the same haplotype (001:01--001:01--036:01) was identified solely in the control population of WHWTs (0 cases; 6 controls). However, this may be due to the WHWT control population being much larger than the case population in that study (43 cases; 166 controls).

Haplotype 2, associated with protection from DM in WHWTs, differs only at the DQB1 locus from haplotype 1, associated with increased risk of DM in Labrador Retrievers, suggesting that the DQB1 locus influences disease susceptibility, or that it is in linkage disequilibrium with other susceptibility loci within the MHC region. Non-synonymous variants in a single locus may influence disease risk by modifying the antigen binding capabilities of the MHC molecules [47], particularly if these variants occur in the hypervariable regions. For example, in human T1D, polymorphisms at positions 56 and 57 of the DQB chain are associated with either disease susceptibility or resistance, according to the amino acid changes in the peptide binding groove [48]. It has been suggested that many risk haplotypes associated with organ-specific autoimmune disease in dogs carry either DQA1*001:01--DQB1*002:01 or DQA1*001:01--DQB1*008:02 haplotypes [42]. The first of these DQ haplotypes is present in haplotype 1 in this study (001:01--001:01--002:01), associated with DM in the Labrador Retriever, however, the second was not present in any haplotypes associated with DM in any of the 12 breeds in the present study. Further examination of the role of these DQ haplotypes in autoimmune disease is warranted, particularly in relation to the effect of variants on the ability of MHC molecules to bind autoantigens and the influence of this on generation of immune tolerance or autoimmunity.

The Cocker Spaniel, a breed with moderate risk for DM [2, 36], is predisposed to autoimmune disease [49–52]. The DLA population profile of Cocker Spaniels is relatively restricted, with most dogs of this breed carrying DRB1*006:01--DQA1*005:01:1 with either DQB1*007:01 or DQB1*020:01 [32]. This restricted DLA profile might be related to a relative hypersensitivity or impaired tolerance towards self-antigen. In the current study, Cocker Spaniels were found to carry one DLA haplotype associated with increased risk of DM and three haplotypes associated with protection from disease (Table 4), suggesting that the MHC plays an important role in determining an individual’s susceptibility to DM in this breed. The DLA haplotype conferring risk, haplotype 6 (006:01--005:01:1--007:01), is the most common haplotype in the breed and is also carried, to some extent, in other dog breeds. In contrast, haplotype 7 (006:01--005:01:1--020:01) confers protection, again implicating the DQB1 locus in disease susceptibility. A previous study of chronic pancreatitis in English Cocker Spaniels also found that the DLA haplotype containing DQB1*007:01 was present in a significantly greater proportion of cases, whereas the haplotype containing DQB1*020:01 was more prevalent in the control population without evidence of pancreatitis [54]. The authors of that study proposed that this suggests a predisposition of English Cocker Spaniels for immune-mediated disease more generally, since DQB1*007:01 has also been implicated in primary immune-mediated haemolytic anaemia [30, 53]. The present study supports this assertion. The DQB1*007:01 allele differs from DQB1*020:01 by 5 amino acids in a hypervariable region of DQB1 exon 2, which has been previously defined [55]. The effect of these amino acid changes on autoantigen binding and presentation by MHC molecules deserves further investigation.

Interestingly, no DLA haplotype associations with DM were identified in the three breeds at highest risk of canine DM: the Samoyed, Tibetan Terrier and Cairn Terrier. It is possible that DM in these breeds is more commonly of a non-immune-mediated aetiology and the MHC plays a less significant role. However, an alternative explanation is that MHC alleles and haplotypes predisposing to DM are not significantly different between cases and controls but are present at high frequency among the breed and exert a ‘fixed breed risk’ rather than individual risk. The possibility of genes, such as the MHC, exerting a fixed risk of DM within certain breeds could be investigated by comparing breeds at high and low risk of DM rather than comparing cases and controls within single breeds. The most common haplotype in the Samoyed breed in this study, haplotype 18 (015:01--006:01--019:01--054:02), was present at high frequency among both cases and controls (63.25% case and 65% control haplotypes) and is exclusively found in this breed. It is possible that this haplotype contributes to the high risk of DM in the Samoyed breed, with other genetic and environmental factors combining to determine whether an individual develops DM. Similarly, six haplotypes were identified exclusively within the Tibetan Terrier breed. Those present at higher frequency (haplotypes 25 and 30) may contribute to the increased risk of DM in this breed.

The two most common haplotypes observed in the study (haplotypes 1 and 6) were also two of the most common identified in the study by Kennedy et al. (2006). This may reflect the fact that Labrador Retrievers and certain terrier breeds (WHWT and Yorkshire Terrier) were highly represented in both studies and a high proportion of dogs in these breeds carry one or both of these haplotypes. Furthermore, these two haplotypes are common in the general dog population (L.J. Kennedy, personal communication).

In the combined analysis with all breeds pooled together, four haplotypes were associated with risk or protection from DM (Table 5). However, three of these were associated with risk or protection in Cocker Spaniels, therefore the combined associations may have been strongly influenced by the haplotype frequencies in this breed. None of the three-locus DLA haplotype associations with canine DM in Kennedy et al. (2006) were replicated in the present study. However, the 2-locus DRB1--DQA1 haplotype associated with risk of canine DM in the previous analysis﻿ (015--006) is present in a protective haplotype (haplotype 16; 015:01--006:01--003:01) in this study. The difference between the two studies is likely to be due to the different number and range of breeds included. Previously, a more heterogeneous population of 87 breeds had been included at lower sample numbers per breed, compared with the current study with a more limited breed profile (*n* = 12 breeds), but each with a minimum of 20 cases and an expanded population of breed-matched controls. The DLA haplotype diversity is known to be restricted within certain breeds but very diverse between breeds [32, 33], therefore altering the relative breed frequencies will impact on the representation of DLA haplotypes in the study population. However, the diverse observations of the two studies highlights how pooling of haplotypes between different breeds can conceal differences among breeds and further emphasizes the need for breed-specific approaches to study the genetics of canine complex disease, such as DM.

There are a number of limitations to the present study which must be considered. The use of sequence-based typing of the MHC has been questioned by a study suggesting that associations found by this method cannot be replicated by a SNP genotyping method [56]. However, SBT allows DNA to be sequenced directly from PCR and the use of specialist software (SBT-Engine, GenDx, Utrecht, the Netherlands) for allele assignment allows reproducibility of results and reduces human error. It is possible that sequencing exon 2 of DLA alleles may have identified associated, but not causative, polymorphisms [57], due to the high degree of linkage disequilibrium in the canine genome. Therefore, follow-up functional studies will be required to determine whether the DLA haplotype associations identified here represent linked or causative polymorphisms. Future work would also benefit from additional samples to allow analysis in a broader range of breeds and a consistent number of cases/controls of each breed. It would also be interesting to carry out pedigree studies in diabetic dogs, considering the MHC haplotypes and disease history of relatives of DM cases. In the future, it may be possible to evaluate an expanded MHC profile (including other genes within this region) from whole genome sequencing and increase the size of the study cohorts through the use of publicly accessible genotyping data for different dog breeds.

The identification of ‘protective’ DLA haplotypes should also be interpreted with caution. In some cases, an allele is present at low frequency in a population, only because a risk allele is present at a relatively high frequency, rather than because it confers protection from disease. This may be the case in Cocker Spaniels in the present study, where there is one dominant ‘risk’ haplotype and the ‘protective’ haplotypes occur at much lower frequency. Larger sample numbers, or exclusion of cases with the dominant risk haplotype in future studies, may help to clarify the importance of the protective associations. Protective MHC haplotypes are documented in human T1D, for example the DRB1*15:01--DQA1*01:02--DQB1*06:02 (HLA-DR2) haplotype which exerts a dominant protective effect in Caucasians [58]. Carriage of this haplotype is highly protective [59], although the effect is not consistent across all ethnic populations and there is some evidence to suggest that the protective effect is modified by other HLA loci [60].

Given the important role of the MHC in the adaptive immune system, the associations identified here support an immune-mediated component to the pathogenesis of disease in some cases of canine DM in some breeds. The associations identified in the Cocker Spaniel, a breed predisposed to autoimmune disease, warrant further follow-up to determine what proportion of diabetic Cocker Spaniels may have an immune-mediated component to the disease. In the DM high-risk breeds, with no DLA associations identified in this study, there remains a high degree of uncertainty surrounding the aetiology of their hyperglycaemia. This once again emphasises the heterogenous nature of canine DM, with different mechanisms leading to beta cell loss and insulin deficiency from breed to breed, and immune-mediated disease playing a role in only a proportion of dogs. In the absence of strong histological or serological evidence of an immune-mediated process being responsible for many cases of canine DM, our data suggest that focus of future investigations in this area should be on breeds where DLA genes do appear to influence disease risk. This could apply to future studies of autoantibodies in canine DM, which previously have not consistently identified circulating autoantibodies [9–12]. In addition, pancreatic necropsy studies in dogs euthanased at or around the time of diagnosis of DM may provide further insight into the histopathological changes in the pancreas during early stages of disease, and could be supported by breed clubs in those breeds where DM is particularly prevalent.

The present findings also emphasise the need for a better understanding of the mechanistic role of canine MHC class II proteins in immunity/immunopathology, particularly in relation how allelic variants associated with disease risk or protection modify the structure and function of MHC molecules, including the peptides they can present. The class II MHC genes confer > 50% genetic risk in human T1D [61] and may also be highly important in immune-mediated forms of canine DM. However, the heterogeneity identified between breeds in this study highlights the need to consider the other genetic and environmental factors which interact to determine an individual’s overall disease risk.

Although these findings are not yet relatable to clinical diagnosis or management of canine DM, they highlight the importance of work to better classify the phenotypes/subtypes of canine DM, similar to the subdivision of human DM patients into T1D, T2D and other forms of disease. This will help to strengthen the power of future analyses by reducing the risk of including disease phenocopies (individuals with the same clinical signs but different pathogenesis to their disease) in study cohorts. Furthermore, a better understanding of how MHC haplotypes influence risk of canine DM may allow stratification of cohorts for clinical research projects according to genotype, which will improve statistical power. In the era of precision medicine, it is possible that canine DM cases where immune-mediated pancreatic destruction can be proven to be responsible for clinical signs may benefit from different management strategies compared to cases with insulin antagonism/resistance or exocrine pancreatic disease.

## Supplementary information

**Additional file 1.**

**Additional file 2.**

## Data Availability

The datasets used and/or analysed during the current study are available from the corresponding author on reasonable request.
