# Supplementary material for: Dog leucocyte antigen (DLA) class II haplotypes and risk of canine diabetes mellitus in specific dog breeds
Source: Canine Med Genet. 2020 Oct 31;7:15. doi: 10.1186/s40575-020-00093-9 (PMC7603736; doi:10.1186/s40575-020-00093-9)
Supplement: Supplementary file 1 — Additional file 1. [file 40575_2020_93_MOESM1_ESM.docx]

**Supplementary Table 1a: Primers used for amplification of DLA-DRB1, -DQA1 and -DQB1 exon 2 (31)**

| Locus | Primer name | Primer sequence |
| --- | --- | --- |
| DRB1 | DRBIn1 | CCG TCC CCA CAG CAC ATT TC |
|  | DRBIn2T7 | TAA TAC GAC TCA CTA TAG GG TGT GTC ACA CAC CTC AGC ACC A |
| DQA1 | DQAIn1 | TAA GGT TCT TTT CTC CCT CT |
|  | DQAIn2 | GGA CAG ATT CAG TGA AGA GA |
| DQB1 | DQB1BT7 | TAA TAC GAC TCA CTA TAG GG CTC ACT GGC CCG GCT GTC TC |
|  | DQBR2 | CAC CTC GCC GCT GCA ACG TG |

The T7 tailed portion is underlined where relevant

**Supplementary Table 1b: Primers used for amplification of DLA-DRB1, -DQA1 and -DQB1 exon 2 in a small number of additional Samoyed samples**

| **Locus** | **Primer name** | **Primer sequence** |
| --- | --- | --- |
| DRB1 (adapted from (59)) | DRBIn1 | CCG TCC CCA CAG CAC ATT TC |
|  | DRB1 M13 REV | TGTAAAACGACGGCCAGTGTCACACACCTCAGCACCA |
| DQA1 (adapted from (60)) | DQA1 M13 FOR | TGTAAAACGACGGCCAGTCTCAGCTGACCATGTTGC |
|  | DQAIn2 | GGA CAG ATT CAG TGA AGA GAG |
| DQB1 (adapted from (61)) | DQB1 M13 FOR | TGT AAA ACG ACG GCC AGT CTC ACT GGC CCG GCC TGT CTC |
|  | DQBR2 | CAC CTC GCC GCT GCA ACG TG |

The M13 tailed portion is underlined where relevant
