# Supplementary material for: Dog leucocyte antigen (DLA) class II haplotypes and risk of canine diabetes mellitus in specific dog breeds
Source: Canine Med Genet. 2020 Oct 31;7:15. doi: 10.1186/s40575-020-00093-9 (PMC7603736; doi:10.1186/s40575-020-00093-9)
Supplement: Supplementary file 2 — Additional file 2. [file 40575_2020_93_MOESM2_ESM.docx]

**Supplementary Table 2: DLA class II haplotype frequencies in dogs with diabetes mellitus and healthy controls for all 12 breeds pooled together**

| **Haplotype ID** | **Haplotype** | | | **Cases**  **n = 1292** | | **Controls**  **n = 1824** | | **Total**  **n** |
| --- | --- | --- | --- | --- | --- | --- | --- | --- |
|  | **DRB1** | **DQA1** | **DQB1** | **n** | **%** | **n** | **%** |  |
| 1 | 001:01 | 001:01 | 002:01 | 176 | 13.62 | 220 | 12.06 | 396 |
| 2 | 001:01 | 001:01 | 036:01 | 31 | 2.40 | 42 | 2.30 | 73 |
| 3 | 001:01 | 009:01 | 001:01 | 58 | 4.49 | 76 | 4.17 | 134 |
| 4 | 002:01 | 009:01 | 001:01 | 67 | 5.19 | 76 | 4.17 | 143 |
| 5 | 005:01 | 003:01 | 005:01 | 9 | 0.70 | 12 | 0.66 | 21 |
| 6 | 006:01 | 005:01:1 | 007:01 | 182 | 14.09 | 199 | 10.91 | 381 |
| 7 | 006:01 | 005:01:1 | 020:01 | 22 | 1.70 | 55 | 3.02 | 77 |
| 8 | 008:02 | 003:01 | 004:01 | 10 | 0.77 | 17 | 0.93 | 27 |
| 9 | 009:01 | 001:01 | 008:01:1 | 77 | 5.96 | 97 | 5.32 | 174 |
| 10 | 009:01 | 001:01 | 008:02 | 27 | 2.09 | 33 | 1.81 | 60 |
| 11 | 011:01 | 002:01 | 013:02 | 9 | 0.70 | 6 | 0.33 | 15 |
| 12 | 011:01 | 002:01 | 013:03 | 38 | 2.94 | 55 | 3.02 | 93 |
| 13 | 012:01 | 001:01 | 002:01 | 1 | 0.08 | 7 | 0.38 | 8 |
| 14 | 012:01 | 004:01 | 017:01-013:03 | 59 | 4.57 | 169 | 9.27 | 228 |
| 15 | 013:01 | 001:01 | 002:01 | 34 | 2.63 | 55 | 3.02 | 89 |
| 16 | 015:01 | 006:01 | 003:01 | 4 | 0.31 | 21 | 1.15 | 25 |
| 17 | 015:01 | 006:01 | 011:01 | 22 | 1.70 | 23 | 1.26 | 45 |
| 18 | 015:01 | 006:01 | 019:01-054:02 | 50 | 3.87 | 91 | 4.99 | 141 |
| 19 | 015:01 | 006:01 | 020:02 | 57 | 4.41 | 76 | 4.17 | 133 |
| 20 | 015:01 | 006:01 | 022:01 | 11 | 0.85 | 14 | 0.77 | 25 |
| 21 | 015:01 | 006:01 | 023:01 | 103 | 7.97 | 128 | 7.02 | 231 |
| 22 | 015:01 | 006:01 | 026:01 | 3 | 0.23 | 15 | 0.82 | 18 |
| 23 | 015:01 | 009:01 | 001:01 | 14 | 1.08 | 14 | 0.77 | 28 |
| 24 | 015:02 | 006:01 | 003:01 | 3 | 0.23 | 6 | 0.33 | 9 |
| 25 | 015:02 | 006:01 | 023:01 | 81 | 6.27 | 134 | 7.35 | 215 |
| 26 | 018:01 | 001:01 | 002:01 | 12 | 0.93 | 19 | 1.04 | 31 |
| 27 | 018:01 | 001:01 | 008:02 | 10 | 0.77 | 12 | 0.66 | 22 |
| 28 | 020:01 | 004:01 | 013:03 | 48 | 3.72 | 49 | 2.69 | 97 |
| 29 | 023:01 | 003:01 | 005:01 | 15 | 1.16 | 18 | 0.99 | 33 |
| 30 | 079:01 | 001:01 | 002:01 | 12 | 0.93 | 21 | 1.15 | 33 |
| **Other haplotypes** | | | | 47 | 3.64 | 64 | 4 | 111 |

Haplotypes identified in dogs with DM and control dogs across all breeds studied. n = number of haplotypes. For ease of reference, haplotype IDs were assigned to the 30 most common haplotypes observed in this study.
